# Preparation and Characterization of Zein-Phosphate Nanoparticles by Nanoprecipitation Method with Potential Use as Fertilizer

**DOI:** 10.1021/acsomega.5c01817

**Published:** 2025-06-13

**Authors:** Milagros Guadalupe Alvarez-Moreno, Francisco Rodríguez-Félix, Carlos Gregorio Barreras-Urbina, Maribel Plascencia-Jatomea, Edgar Omar Rueda-Puente, Juan José Reyes-Pérez, José Agustín Tapia-Hernández, Silvia Elena Burruel-Ibarra, Tomás Jesús Madera-Santana, Itzel Yanira López-Peña, Josué Elías Juárez-Onofre, Irela Santos-Sauceda

**Affiliations:** † Departamento de Investigación y Posgrado en Alimentos, 27813 Universidad de Sonora, Blvd. Luis Encinas J, Calle Av. Rosales &, Centro, 83000 Hermosillo, Sonora, Mexico; ‡ Centro de Investigación en Alimentación y Desarrollo, Carretera Gustavo Enrique Astiazarán Rosas, No. 46, CP. 83304 Col. La Victoria, Mexico; § Departamento de Agricultura y Ganadería, Universidad de Sonora, Carretera 100 a Bahia de Kino km. 21.5, 83000 Hermosillo, Sonora, Mexico; ∥ 27910 Universidad Técnica Estatal de Quevedo, Av. Carlos J. Arosemena 38, 91050 Quevedo, Ecuador; ⊥ Departamento de Investigación en Polímeros y Materiales, Universidad de Sonora, 83000 Hermosillo, Sonora, Mexico; # Departamento de Investigación en Física, Universidad de Sonora, 83000 Hermosillo, Sonora, Mexico

## Abstract

The design of novel alternatives aimed at promoting sustainable agriculture is essential for counteracting the significant demand to feed an ever-growing population. The amphiphilic properties of zein make it an excellent biomaterial for the development of prolonged-release fertilizer. The objective of this work was to prepare and characterize phosphate (Ca­(H_2_PO_4_)_2_·H_2_O) release particles based on zein that can be used in agriculture to reduce the loss of this nutrient through runoff and leaching. Zein nanoparticles were prepared by nanoprecipitation method using ethanol (70% v/v). A 3 × 3 × 3 factorial design was used for phosphate-loaded zein nanoparticles. The zein-phosphate nanoparticles with zein 3% (w/v), poloxamer 188 0.15% (w/v), and phosphate 1 and 2% (w/v) were significantly the smallest in size (367 ± 16 nm and 326 ± 15 nm, respectively), the polydispersity index for both concentrations of Ca­(H_2_PO_4_)_2_·H_2_O was 0.3 ± 0.1, and there was a high z-potential on both nanoparticles (79.8 ± 11.6 mV and 178.8 ± 9.9 mV for nanoparticles with 1 and 2% w/v of Ca­(H_2_PO_4_)_2_·H_2_O). Fourier Transform Infrared Spectroscopy analysis shows that hydrogen bonds are the mechanism by which the PO_4_^–3^ groups are joined to the nanoparticles, and the deconvolution of the amide I shows the beta sheets are the predominant secondary structure for nanoparticles with 2% phosphate. Finally, the release of phosphate was close to 20%. In this work, it was possible to find favorable conditions for the formation of zein nanoparticles loaded with monobasic phosphate monohydrate by nanoprecipitation.

## Graphical abstract

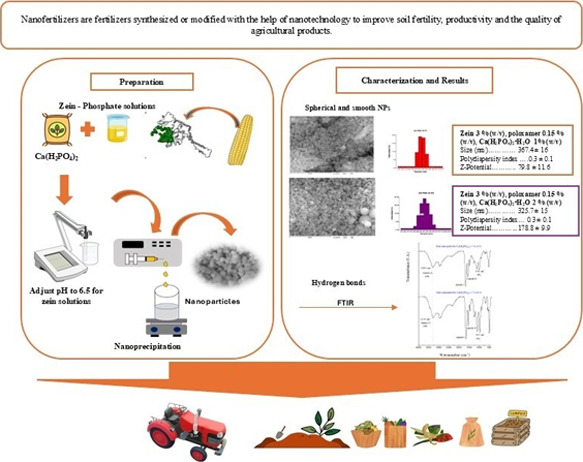

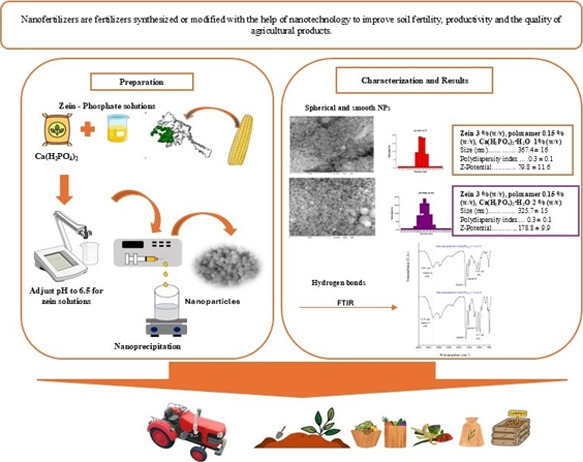

## Introduction

Zein is a prolamin of corn. Corn is a crop of global importance due to its diversity of uses. In general, it can be subjected to three types of processes: (1) dry milling, for human food use; (2) wet milling, for ingredients and industrial inputs (nonfood); and (3) bioethanol production, in addition to the forage being used as feed for livestock.^1^ It is possible to obtain zein from wet milling and bioethanol production processes. In general, corn grains are rich in protein, as they have a high amount of zein. However, it is deficient in essential amino acids and does not offer a significant nutritional value to humans. More than 50% of zein amino acid residues are hydrophobic, being insoluble in water^2^ but soluble in organic solvents such as ethanol and propylene glycol.^3^ On the other hand, it has great potential in industry due to its amphipathic, self-assembly,^4,5^ biodegradable, and renewable properties.^6,7^ Due to these properties, zein has a wide field of application including drug,^8^ nutrient,^9^ pesticide,^10^ environmental remediation,^11^ and active compound^12^ delivery systems like slow release fertilizers.

On the other hand, phosphorus (P) is one of the most limiting macronutrients for productivity in crops.^13^ Soil nitrogen and phosphorus can maintain a positive relationship because the interaction of these nutrients can increase the solubility of phosphorus through nitrification processes in alkaline soils.^14^ However, nearly 80% of the applied phosphorus via conventional fertilizers can be lost to the environment through lixiviation, precipitation reactions,^15,16^ and surface runoff.^17^ Lixiviation leads to the eutrophication phenomenon caused by the high concentrations of dissolved phosphorus in water promoting algae growth due to the decrease of oxygen levels in water, adversely affecting surrounding organisms.^18,19^ Meanwhile, precipitation reactions depend directly on soil pH.^20^ The presence of iron and aluminum oxides and hydroxides in acid soils can form aggregates with phosphorus and immobilize its assimilation by plants.^21^ The phosphate groups can react with calcium ions (Ca^2+^) and magnesium ions (Mg^2+^) in alkaline soils.^22^ Therefore, new alternatives are necessary for agricultural soils to increase the availability of phosphorus for a long time. Based on these circumstances, it is necessary to promote alternatives that improve the use of phosphorus in agricultural crops.

To mitigate the negative effects of conventional fertilizer, the use of prolonged-release fertilizers has been suggested.^23,24^ Prolonged-release fertilizer is defined by the American Association of Plant Food Control Officials (AAPFCO) as a prolonged-release system that contains plant nutrients in a form that delays their release availability for adsorption and plant use after application.^25^ Biopolymer-based phosphorus nanofertilizers are an alternative to conventionally used fertilizers.^26^ A Biopolymer is a molecule with repetitive units and isolated from natural sources that can be nucleic acids, proteins, or carbohydrates. Biopolymers have made possible the development of emerging, environmentally friendly materials.^27,28^ Some of the biopolymers more commonly used like slow-release systems are chitosan,^29^ starch,^30^ cellulose,^31^ gelatin,^32^ collagen,^33^ zein,^34^ pectin,^35^ nanocomposites,^36^ and others.^37^ The elaboration of the particles is necessary for the subsequent encapsulation of the phosphorus. When selecting a biopolymer, it is important to consider its possible response to environmental conditions, such as pH, temperature, moisture, or soil enzymes. In general, hydrogels based of biodegradable polymers are used, such as cellulose for systems sensible to moisture^38^ or carrageenan for systems sensible to pH changes.^39^ Qi et al.,^34^ found positive results by developing a slow-release fertilizer made of phosphorus enveloped in a zein matrix. In said experiment, it was observed that soybeans are grown under greenhouse conditions. Therefore, it is possible to affirm the potential use of a zein matrix in agricultural crops.

According to the model of Wang and Padua,^5^ the formation of zein nanoparticles is due to their capability to self-assemble and their amphiphilic property, which can occur in three steps. In the first phase, as ethanol evaporates, the polarity of the reaming solvent gradually increases, thereby inducing a structural transition in the secondary structure from α-helix to β-sheet, driven by the hydrophobic characteristics of zein. Second, as evaporation continues, the β-sheets will pack antiparallel due to the hydrophobic interactions of two adjacent β-sheets. Finally, the band of β-sheets begins to coil into a ring shape, until the central hole closes. In this way, the zein sphere has been formed (Figure 1).^5^

**Figure 1:**
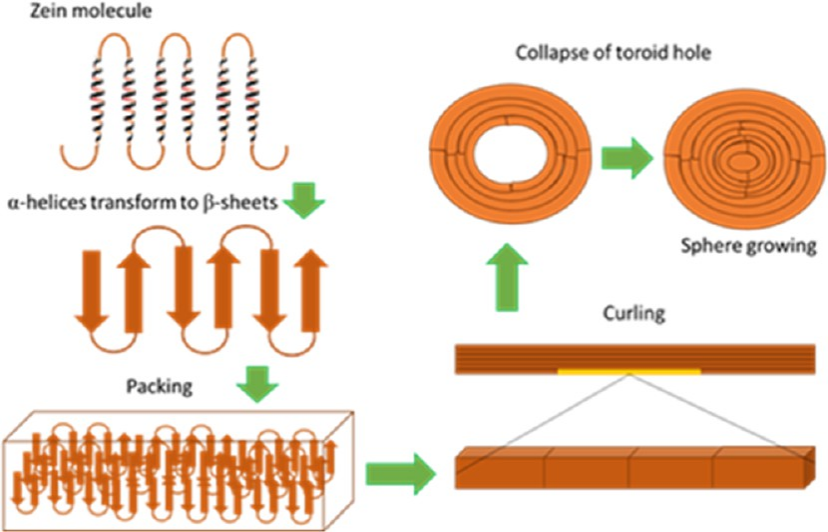
Proposed mechanism for the obtention of zein nanoparticles after evaporation-induced self-assembly. Adapted in part with permission from Wang, Y., & Padua, G. W. (**2012**). Nanoscale characterization of zein self-assembly. Langmuir, *28*(5), 2429–2435. Copyright 2025 American Chemical Society.^5^

The use of prolonged-release fertilizers has some advantages over conventional fertilizers such as fewer losses of fertilizer in crops, greater yield production, and lower long-term costs, representing an economic benefit. Additionally, reducing fertilizer loss during cultivation decreases the ecological impact. Finally, as the plant uses nutrients to a greater extent, the quality of the product increases, and there is a social benefit.^23,24^ Slow-release fertilizers account for about 16.8% of the total global fertilizer market value.^40^ However, the costs of slow-release fertilizers are often higher than those of conventional use.^24^ Prices for polymer-coated fertilizers can be up to three times more expensive,^41^ but its cost has decreased over the past few years, especially in the Chinese market where they are considerably lower than in other markets. The price of polymer-coated slow-release fertilizers in 2014 ranged from 700 to 1500 dollars per ton, while in 2020 it ranges from 350 to 600 dollars,^42^ accounting for 62.9% of the market.^43^ The difference in price within the same year is due to the covering material and the complexity of the manufacturing process.^40^

Nanofertilizers with metallic nanoparticles can induce physiological, biochemical, and genetic changes in plants, increasing their growth and yield, as well as the production of bioactive compounds.^44^ This contributes to the generation of higher-quality food and the promotion of more sustainable agricultural practices. The use of biopolymer-based nanofertilizers with metallic nanoparticles has been widely used in the literature in various crops and their effects have been studied, it has been observed that the use of Cu, Zn, Se, or Si is capable of increasing quality parameters of the crop and a higher concentration of phenolic compounds in Capsicum annuum and Avena sativa L.^45,46^ It has also been reported that the use of biopolymer-based nanofertilizers can stimulate defense mechanisms and impacts plant growth and development^47^ and increase in growth and weight.^48^ In general, recommendations involve the usage of low doses (10 mg kg^–1^ of soil) of metallic nanoparticles in order to avoid adverse effects such as oxidative stress^49^ and the use of biopolymer-based coatings that can regulate the dissolution and loss of nutrients.^50^ However, it has been reported that this type of nanofertilizer tends to translocate into plant tissue^51^ and can affect the soil microbiota due to their antibacterial properties,^52^ for this reason the use of metallic nanoparticles can be controversial.

Nanofertilizers based on biopolymers are mainly made from polysaccharides and proteins and have shown good results. Van et al.^53^ found an increase in chlorophyll content and increased nutrient input using chitosan nanoparticles in Robusta coffee, while Dhlamini et al.^54^ observed an increase in plant height and leaf number using chitosan-based nanofertilizers. Salinas et al.^55^ used a nanofertilizer based on zein nanoparticles and observed an increase in chlorophyll concentration and root length in soybean crops. Favorable results have also been observed in chitosan-based NPK nanofertilizers, with an improvement in nutrient uptake and chlorophyll content in coffee beans crops^56^ and an increase in leaf area, root length, and water content.^57^ Other authors,^58^ studied the effect of a slow-release phosphate fertilizer on microbial communities and enzymatic activity in soil and have found favorable results. On the other hand, Reis et al. (2022)^15^ tested three types of slow-release phosphorus fertilizers where triple phosphate (the same one used on current research) showed the best results in crops. In addition to nanofertilizers, nanopesticides^59^ and biosensors^60^ have also been introduced to reduce product losses or monitor important changes. However, despite the visible advantages of using nanofertilizers, it is important to remember that there is still limited knowledge about biosafety, adverse effects, fate, and biological reactivity in the environment.^61^ Therefore, toxicity studies are necessary before introducing and testing new materials into a crop.

Nowadays, there are different techniques to produce prolonged- release fertilizers,^23,62^ among which the nanoprecipitation method stands out.^63^ This method is based on nucleation theory. Nucleation occurs when the concentration of the polymer exceeds the critical saturation limit, causing the breakdown of the interface between the polymer and the solvent upon addition to the aqueous phase and initiating the formation of nanoparticle nuclei. The nucleus growth occurs by condensation or coagulation of the suspended particles accompanied by energy release. Particle aggregation occurs after growth and is influenced by polymer and surfactant concentrations as well as temperature. During this step, control is maintained by agitation to uniformly homogenize the formed nanoparticles. This method can be used for various materials, so it is possible to load a wide range of organic or biological molecules, and these particles can be used in the prolonged-release system.^64^

The preparation of zein nanoparticles by flash nanoprecipitation^65^ and nanoprecipitation controlled by pH has been previously reported.^66^ In both cases, parameters such as solute concentration, solvent, and flow rate were evaluated for their influence on the particle size. To recognize the mechanisms of action, as well as the impacts of fertilizers in the agricultural environment, it is important to develop more sustainable ways to apply them. The objective of this study was to develop and characterize a zein-based phosphorus (Ca­(H_2_PO_4_)_2_·H_2_O) prolonged-release system by the nanoprecipitation method. The use of biopolymers, in this case, zein like a matrix of the system to encapsulate Ca­(H_2_PO_4_)_2_·H_2_O, replaces the use of synthetic polymers. Until now, the use of synthetic fertilizers could cause environmental damage (salinization, greenhouse gas generation, eutrophication, etc.).^67^ A zein-based nanofertilizer is a more sustainable and nontoxic alternative. In addition, the size of the nanoparticles formed by biopolymers is large enough to avoid the oxidative stress generated by much smaller nanoparticles in the soil microbiota.^68^

## Materials

The zein used was from Sigma-Aldrich (EC number: 232-722-9, lot # SLCL4585), ethanol from Jalmek (lot # 22260815E53), monobasic calcium phosphate monohydrate (Ca­(H_2_PO_4_)_2_·H_2_O), (lot # BCCB0891, purum p.a. ≥ 85%), and poloxamer 188 (lot # BCBM1662 V) from Sigma-Aldrich.

## Methods

### Preparation of Zein Solutions

Three solutions of zein at 2, 3, and 4% w/v, in ethanol at 70% (v/v) and pH of 6.5 were prepared. A previous zein-ethanol solubility study was carried out, in which the use of 70% (v/v) ethanol was determined as the most suitable percentage. For the preparation of zein solutions, the solubility in ethanol and the effect of pH during nanoparticle formation was considered.

### pH Measurements

The pH value of the solutions was determined by a potentiometer (HI-2221, Hanna Instruments).

### Density (ρ)

The density was determined by the method reported by Tapia-Hernández et al.,^69^ which consists of the use of pycnometers. Pycnometers were previously placed in an oven at 100 °C for 1 h and subsequently in a desiccator for 1 h to ensure achievement of a constant weight; then, they were weighed on an analytical balance (OHAUS, PX Series Balances). ρ was calculated according to eq 1, where *M*1, *M*2, and *M*3 represent the weight of the empty pycnometer, the weight of the pycnometer with water, and the weight of the pycnometer with the problem solution, respectively. The measurements were taken in triplicate.

1 $$\rho =\frac{M3-M1}{M2-M1}1g/{cm}^{3}$$

### Elaboration of Nanoparticles

The zein spheres were synthesized by the nanoprecipitation method described by Fessi et al.^70^ with some modifications. The solutions of 0.05, 0.1, and 0.15% (w/v) of poloxamer 188 and, 2, 3, and 4% (w/v) of zein was used on experimental design using a 3 × 3 factorial design (Table S1). 5 mL of the organic phase and 5 mL of the aqueous phase were prepared. The organic phase consisted of a zein solution at pH 6.5 in ethanol 70% (v/v), which was added using a peristaltic infusion pump (model TL-F6, TonguTech, China) and the aqueous phase was a solution of the surfactant Poloxamer 188 (2-(2-propoxypropoxy) ethanol) at different concentrations in water. The aqueous phase was kept under constant agitation at 100 rpm using a magnetic stirrer (Model C-MAG HS 7 S1). To obtain spheres loaded with phosphorus, the previous procedure was carried out with the addition of calcium phosphate monobasic monohydrate (Ca­(H_2_PO_4_)_2_·H_2_O) (1, 2 and 3% w/v) at the aqueous phase and experimental design used was 3 × 3 (Table S2).

### Atomic Force Microscopy

The Scanning Pro Microscope (JSPM-4210, Jeol, Japan) is equipped with a MikroMasch NSC15/NO AL cantilever with a nominal spring constant of 40 N/m and a resonance frequency of around 325 kHz. Images were obtained in noncontact mode. The zein nanoparticles were placed in the corresponding sample holders, where the solvent was evaporated at 25 °C.

### Drying of the Samples

Zein nanoparticles at 2, 3, and 4% (w/v) with 0.1% (w/v) poloxamer 188 were subjected to the following drying methods: (1) oven with a vacuum pump (Yamato, model JAA11X04, Japan), at 50 °C and −65 kPa for 6 days; and (2) lyophilization (Labconco Freezone 6, Kansas City, MO, USA).

### Scanning Electron Microscopy

For morphological characterization, a scanning electron microscope (JEOL 7800F brand, model 5410LV scanning electron microscopy (SEM), Pleasanton, CA, USA) at a voltage of 15 kV will be used. The samples were solid-analyzed and plated with gold as a conductive material before their morphological characterization. The images obtained were observed at magnifications of 10k× for the nanoparticles obtained from concentrations of 2, 3, and 4% (w/v) of zein.

### Dynamic Light Scattering and Z-Potential Measurements

The size and z-potential were determined by zeta sizer nano equipment (Malvern Panalytical, model Nano-ZS90). For each measurement in dynamic light scattering (DLS), 3 mL of suspension of each of the concentrations of the experimental design were used. The concentration used was 1 mg/mL. The light diffraction index for the phosphor-loaded nanoparticles was 1.360, read in sextuplicate at an angle of 90°, in a 70% (v/v) ethanol solution with deionized water. For the determination of the z-potential, the Smoluchowski equation was used.

### Rheology

The rheological behavior of zein nanoparticle solutions at 3% (w/v), with 0.15% (w/v) poloxamer, and with 1 and 2% (w/v) monobasic phosphate monohydrate was analyzed. The freshly prepared samples were analyzed using a rheometer (model MCR102, Anton Paar, Germany), with a concentric cylinder. The cutting speed used was in the range of 1 to 300 s^–1^ at 25 °C.

### Fourier Transform Infrared Spectroscopy

FT-IR equipment (PerkinElmer, Subtech Spectrum model, USA) was used with an Attenuated Total Reflectance attachment (ATR), using a diamond detector. A wavenumber range of 4000–400 cm^–1^ will be used, with a resolution of 16 scans per spectrum. The spectra were plotted with the help of the OriginPro8 program. For the determination of the second derivative of the original spectrum (deconvolution), it was made from the relative area of each of the zein amide I bands with Ca­(H_2_PO_4_)_2_·H_2_O trapped.

### Differential Scanning Calorimetry

The determination was carried out on nanoparticles with and without phosphorus in a Differential scanning calorimeter (PerkinElmer, model 8500, Shelton, CT, USA), at a heating rate of 10 °C/min/min under a nitrogen atmosphere. The thermograms were plotted with the help of OriginPro 8 software.

### Prolonged Release of Ca­(H_2_PO_4_)_2_·H_2_O from Zein Nanoparticles

From the nanoparticles synthesized with 2% of Ca­(H_2_PO_4_)_2_·H_2_O, a pellet with dimensions of 1.3 cm × 0.3 cm and a weight of 0.499 g was used, which contained a concentration of 0.2 g of Ca­(H_2_PO_4_)_2_·H_2_O. The pellets were placed in a beaker with 1 L of deionized water that was kept under constant stirring at 100 rpm on a stir plate. Aliquots of 1 mL were taken at a time interval of 0 to 12 h. The concentration of nutrients in the water was determined using a UV–vis spectrophotometer (model Varian Cary 50 Conc, USA). Concentration measurements were performed in triplicate. The absorbances obtained were then extrapolated to the calibration curve to determine the percentage of release.

### Statistical Analysis

A two-way ANOVA was performed to determine the interaction between the percentage of zein and ethanol and its effect on solubility and the DLS analysis. A one-way ANOVA for density, zein concentrations, and pH was performed with a subsequent Tukey test using the InfoStat version 2020 statistical software. SEM, FTIR-ATR, and differential scanning calorimetry (DSC) analyses were interpreted according to the literature, and the graphs were obtained using Origin Pro 8.

## Results and Discussion

### Preparation of Zein Solutions

No significant differences were found between the zein concentrations (2%, 3%, and 4% w/v) and the ethanol–water concentrations used (between 70 and 90% (v/v)) in the solubility tests on the solutions of zein-ethanol–water. Zein is insoluble in water and absolute ethanol but soluble in ethanol–water mixtures. However, the 70% (v/v) ethanol–water concentration was selected to prepare the nanoparticles because it has been previously reported that, from this concentration, it is possible to form nanoparticles with greater solubility.^5,71^ Other authors^2,72^ agree that an increase in ethanol concentration can increase particle size, this has been seen specifically at concentrations greater than 70% (v/v) in ethanol solution. In addition, ethanol mixtures greater than 90% (v/v) tend to decrease protein solubility and separate the phases^73^ and some authors reported the maximum solubility of commercial zein has been reported at 70–80% (v/v).^74^ Chen et al.^2^ explained this behavior due to a high concentrations of ethanol in the solution, which induce the exposure of the sulfhydryl group to the surface during protein unfolding, and in turn exposes the nonpolar amino acids of the protein, affecting its native conformation and reducing its solubility. Therefore, the medium becomes too hydrophobic, which reduces interactions with the polar groups, and consequently the zein precipitates or forms aggregates.

### pH Measurements

The pH obtained in zein solutions is around 5. The acidic pH is due to the high proportion of glutamic acid in the protein (20%), because it is a proton donor and is also a hydrophilic amino acid, which is why it is oriented on the outside of the protein. Within the protein, there are also polar glutamine residues that form hydrogen bonds between α-helix.^75^ However, the pH of the zein solutions used for the formation of nanoparticles was modified to 6.5, because managing a pH of 6.5 could help generate repulsive electrostatic charges between the terminal groups of glutamic acid of the zein. The increase in pH in the solution may be since the pH of the aqueous phase is slightly alkaline; therefore, upon contact with the organic phase (pH 6.5), it increases to a pH of 7.5 (Table 1). Said behavior can be explained due to a change in the dielectric constant of the medium when the zein solution (organic phase) came into contact with the poloxamer 188 solution (aqueous phase), which is slightly basic and increased the pH of the nanoparticles. In addition, because the ethanol solution has a lower dielectric constant (which causes greater electrostatic interactions), that is, less capacity to separate ions of opposite charges than the aqueous solution (water has a higher dielectric constant, which decreases electrostatic interactions), which allows greater ionization of the functional groups and shifts the pH. The protein in solution is above its isoelectric point (6.2), which causes the carboxyl groups to be deprotonated and the protein to be negatively charged.^76^ Therefore, the negative charges of the protein on the surface promote hydrophobic protein–protein and water–protein interactions, resulting in the formation of small particles stabilized by electrostatic interactions.^75^

**Table 1:** pH of Nanoparticles in Different Concentrations^a^

zein % (w/v)	surfactant % (w/v)	pH
2	0.05	7.6 ± 0.10^B^
2	0.1	7.4 ± 0.04^A^
2	0.15	7.5 ± 0.06^AB^
3	0.05	7.3 ± 0.03^A^
3	0.1	7.4 ± 0.12^AB^
3	0.15	7.4 ± 0.08^AB^
4	0.05	7.5 ± 0.09^AB^
4	0.1	7.4 ± 0.06^AB^
4	0.15	7.4 ± 0.09^AB^

a Measurements were made in triplicate. Different letters indicate significant differences.

The influence of pH on the formation of nanoparticles has been described by Podaralla and Perumal.^66^ The authors suggest that nanoparticles have a higher formation capacity when the pH is above the isoelectric point. The pH obtained in the zein solutions was around 5, which suggests that the protein is in cationic form because its isoelectric point is pH 6.0. This is mainly due to the low amount of basic amino acids, which suggests that the predominant form of the protein is the cationic form.

The effect of factors such as ethanol and pH on the formation of zein nanoparticles by nanoprecipitation can be related to the interaction of the ethanol molecule and the protein in the equality of charges caused by hydronium (H_3_O^+^) and hydroxyl (OH^–^) ions of the basic and acid media that were used.^77^ Another of the influences of pH on the stability of the formation of nanoparticles is that ethanol could function as a surfactant since the hydrophobic part of the molecule interacts with the surface of the protein, which would form a protective layer.^78^

### Density

The density, as well as parameters such as viscosity and diffusivity of an ethanol/water binary solution are dependent on the ethanol ratio.^72^ The density is the ratio of mass to the volume.^79^ Significant differences were observed in the density due to the zein concentrations (*F*(6) = 633.9, *p* < 0.0001; Figure 2). As can be observed in Figure 2, the density of the zein solution increased as the amount of zein was increased in the ethanol/water mixture.

**Figure 2:**
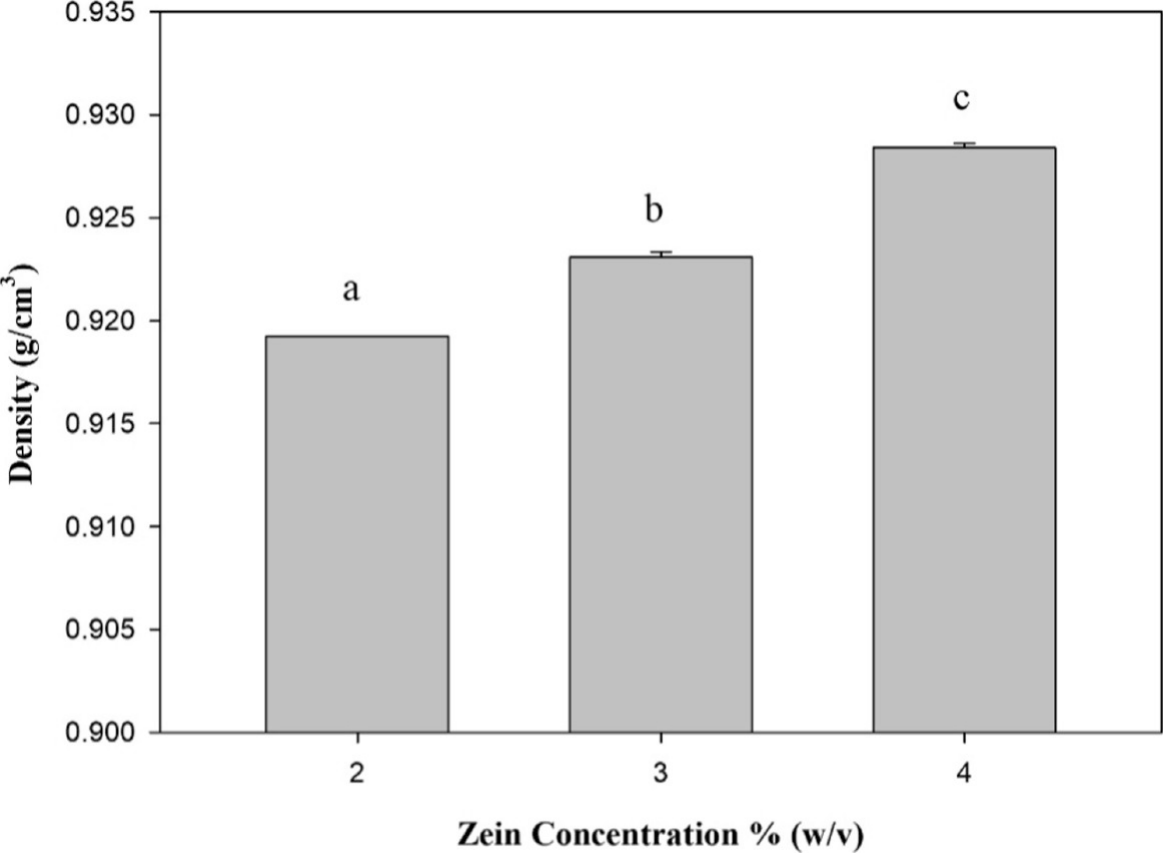
Effect of zein concentration (w/v) on the density of a 70% (w/v) zein-ethanol solution. The figure shows the means of the density determinations made in mean ± their standard deviation (*n* = 3). Different letters represent statistically significant differences.

### Atomic Force Microscopy

This technique shows the morphology of the nanoparticles. In all cases, zein nanoparticles were observed, showing spherical shapes with sizes in the nanoscale (Figure 3). The methodology was realized with the goal of observing the morphology of zein nanoparticles before the drying method and ensuring their synthesis. However, the morphology was affected by the drying method of the samples (Figure 3).

**Figure 3:**
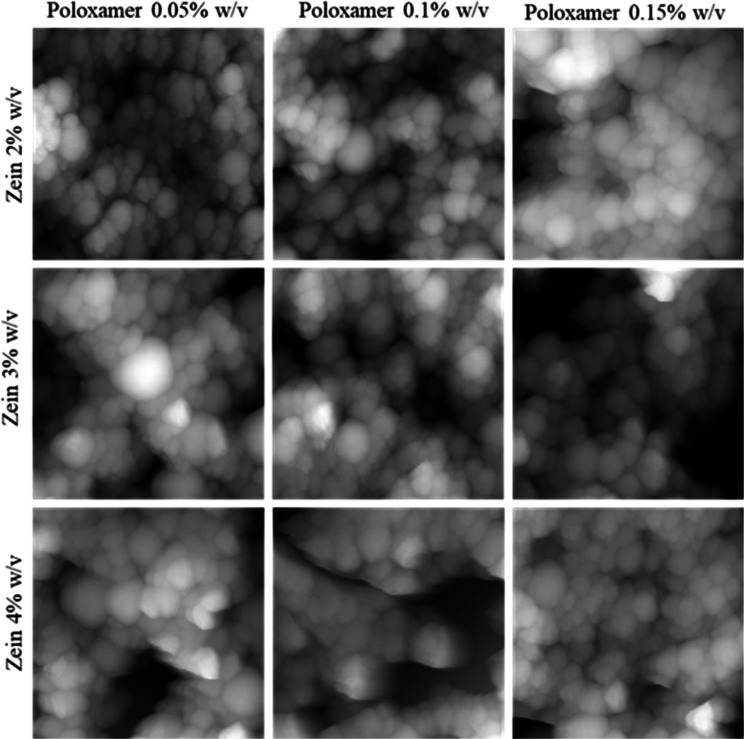
Effect of zein and poloxamer 188 concentrations on the morphology of zein nanoparticles observed by AFM.

### Scanning Electron Microscopy

The samples processed in the different drying methods at concentration of 2, 3, and 4% (w/v) of zein and 0.05, 0.1, and 0.15% (w/v) of poloxamer 188 presented morphological variations that are observed in Figures 4 and 5. It is possible to observe that the nanoparticles dried in a vacuum oven have almost completely lost their morphology, while the lyophilized ones preserve it to a greater extent. The most relevant data for each method are described below.

1 Oven with a vacuum pump at 50 °C and −65 kPa for 6 days. At the end of drying, a dry mass adhered to the container, that could be easily removed, was obtained. Figure 4 shows images of zein particles by SEM adhering to each other at concentrations of 2 and 3% (w/v); however, the particles have completely disintegrated at higher concentration. These changes in nanoparticles can be explained due to the fact that zein is a protein with a tertiary structure stabilized by noncovalent interactions, such as hydrogen bonds and hydrophobic forces.^80^ The effect of temperature (50 °C) on the tertiary structure causes these types of interactions to weaken, cause protein denaturation, and directly affects the structural integrity of the nanoparticles.^80^ Sun et al., (2016) proposes a three-stage mechanism on how heat treatment affects the physical and structural properties of zein, which consists of folding, unfolding, and aggregation of the protein.^81^ In addition, the combined effect of the medium (70% v/v ethanol solution) and temperature alters the hydrophobic interactions that stabilize the nanoparticles.^82^
2 Lyophilization. This method was the most effective with respect to the drying time of the nanoparticles. The ethanol in the samples was previously evaporated in a laminar flow hood and subsequently introduced into the freeze-dryer for 2 days. At the end of the drying time, the samples presented the appearance of a very fine, whitish powder. Figure 5 shows that the images of the samples dried by this method show more stability, even at the highest concentration, being at 3% (w/v) where the largest size (but also the greatest stability of the particles) was presented.

**Figure 4:**
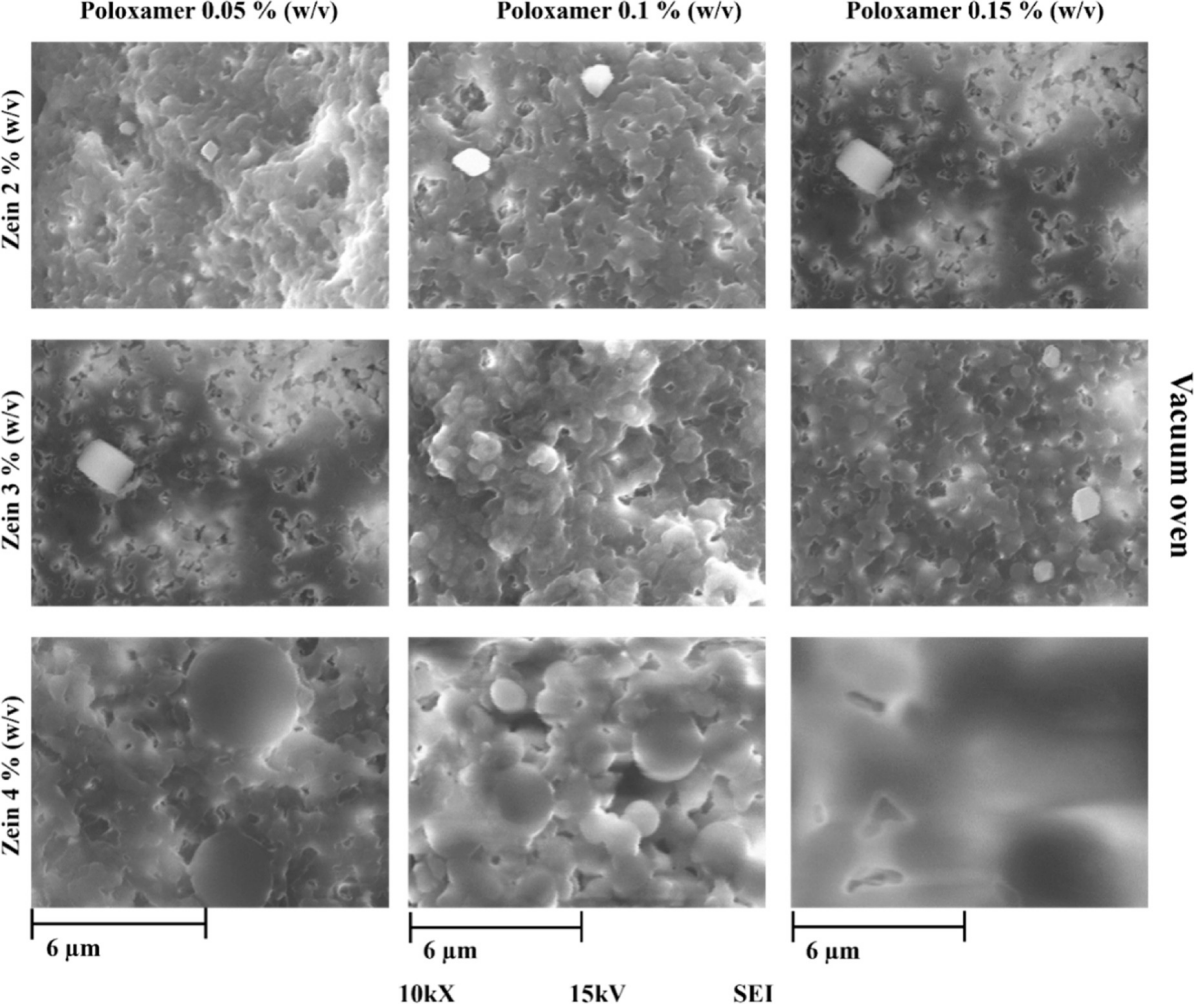
Zein nanoparticles in different concentrations of zein and poloxamer 188 dried by vacuum oven at 10 000× magnification and 15 kV by SEM.

**Figure 5:**
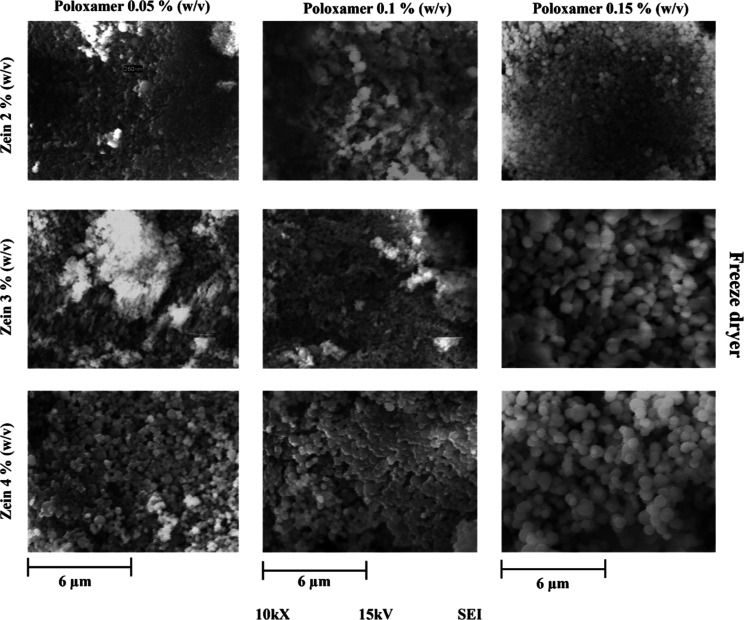
Zein nanoparticles in different concentrations of zein and poloxamer 188 dried by freeze-dryer at 10 000× magnification and 15 kV by SEM.

Figure 4 shows the samples dried by vacuum oven. It is possible to observe that at higher zein concentrations, the morphology is lost until a deformed mass is left, which makes it impossible to determine its size. The loss of morphology could be due to an affectation of the various layers of the nanoparticle cover, where, due to the drying process, the proteins could reorganize or accumulate, causing a deterioration in their morphology.^83^ Figure 5 shows the samples lyophilized. It was observed that the higher the concentration, the larger the size of the nanoparticles, especially on zein 4% (w/v) and 0.15% (w/v) of poloxamer 188, and similar results have been reported by other authors.^84,85^

Proper drying of the samples is required to preserve their morphology as best as possible. This will help preserve its properties, such as surface contact and encapsulation ability. The importance of this step lies in the fact that the zein nanoparticles in solution lose their stability over time. Around 2 weeks after being produced, aggregates began to be observed, and after 3 weeks, the aggregates of zein nanoparticles could not be dissolved.

Even though in both drying processes, tensions are created that can facilitate the aggregation, denaturation, or chemical degradation of the proteins,^86^ freeze-drying was the most suitable for obtaining nanoparticles (Figure 5). The importance of nanoparticles being lyophilized after being synthesized lies in the fact that water is an active participant in many chemical degradation processes of proteins. Therefore, the longer they remain in suspension, the more prone they are to degradation, unless the liquid formulation is stabilized; otherwise, lyophilization is the best option.^87^ Poloxamer 188 helps to stabilize the nanoparticles formed since it acts as a steric hindrance that prevents protein–protein aggregation during lyophilization and thus prevents its surface area from decreasing.^86^ Therefore, lyophilization is the most appropriate method to maintain the morphology of the nanoparticles.

### Dynamic Light Scattering

Nanoparticles with and without phosphorus were analyzed by this method to determine particle size, polydispersity index, and z-potential. This methodology makes it possible to determine the physical characteristics of particles in suspension through Brownian motion.^88^

Freeze-dried zein nanoparticles. The size of the synthesized particles differed from the concentrations of zein (w/v) (*F*(2) = 4.78, *p* = 0.0125) and poloxamer (w/v) (*F*(2) = 5.23, *p* = 0.0087) used. The size mainly determines whether it is nanoparticles or microparticles. Polymer nanoparticles are defined as solid, colloidal particles in the range of 10–1000 nm.^89^ The mean sizes of the concentrations of 2, 3, and 4% (w/v) of zein were 692 ± 335, 1125 ± 810, and 761 ± 444 nm, respectively (Figure 6). Therefore, the concentration at 3% (w/v) of zein are microparticles, while concentrations at 2 and 4% (w/v) are nanoparticles. There are no significant differences between the concentrations of 2 and 4% zein, while the size difference observed at 3% (w/v) zein is due to the size obtained when the sample was synthesized with 0.15% poloxamer 188 (Figure 6). The means of the 0.05 and 0.1% (w/v) poloxamer concentrations were significantly different at the 0.15% (w/v) concentration of poloxamer, which was where the largest mean size was presented (1155 nm) compared to the other concentrations that did not; they presented a significant difference between them (635 nm for 0.05% and 658 nm for 0.1%). Zhong and Jin,^77^ reported a positive correlation between zein concentration and nanoparticle diameter. However, no relationship was observed between zein concentration and nanoparticle size; this may be because there are no significant differences in the viscosities of the solutions (Table 5). Based on these results, the higher concentration of poloxamer 188 in the solution can produce larger nanoparticles. It has been shown that particle size can influence the coagulation mechanisms; nanoparticles are susceptible to self-aggregation due to Brownian movement, which is why the use of a surfactant that works as a steric hindrance is most recommended and can prevent such aggregation. On the other hand, microparticles are more susceptible to sedimentation due to their higher molecular weight; however, they tend to be more stable and disperse more easily.^90^

**Table 2:**
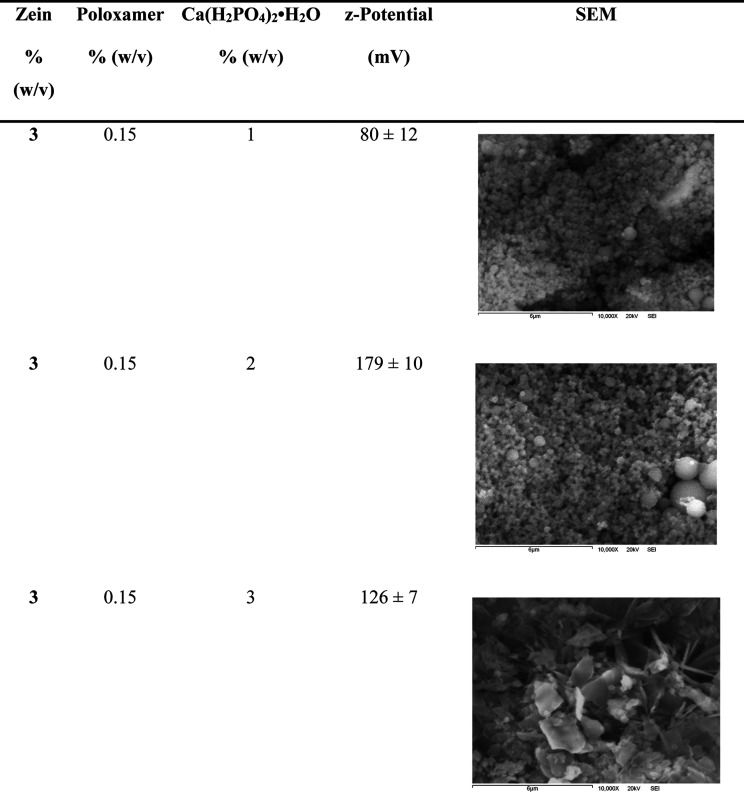
SEM Micrographs on 10,000× of the Most Stable Phosphorous Zein Nanoparticles Based on High Z-Potential

**Figure 6:**
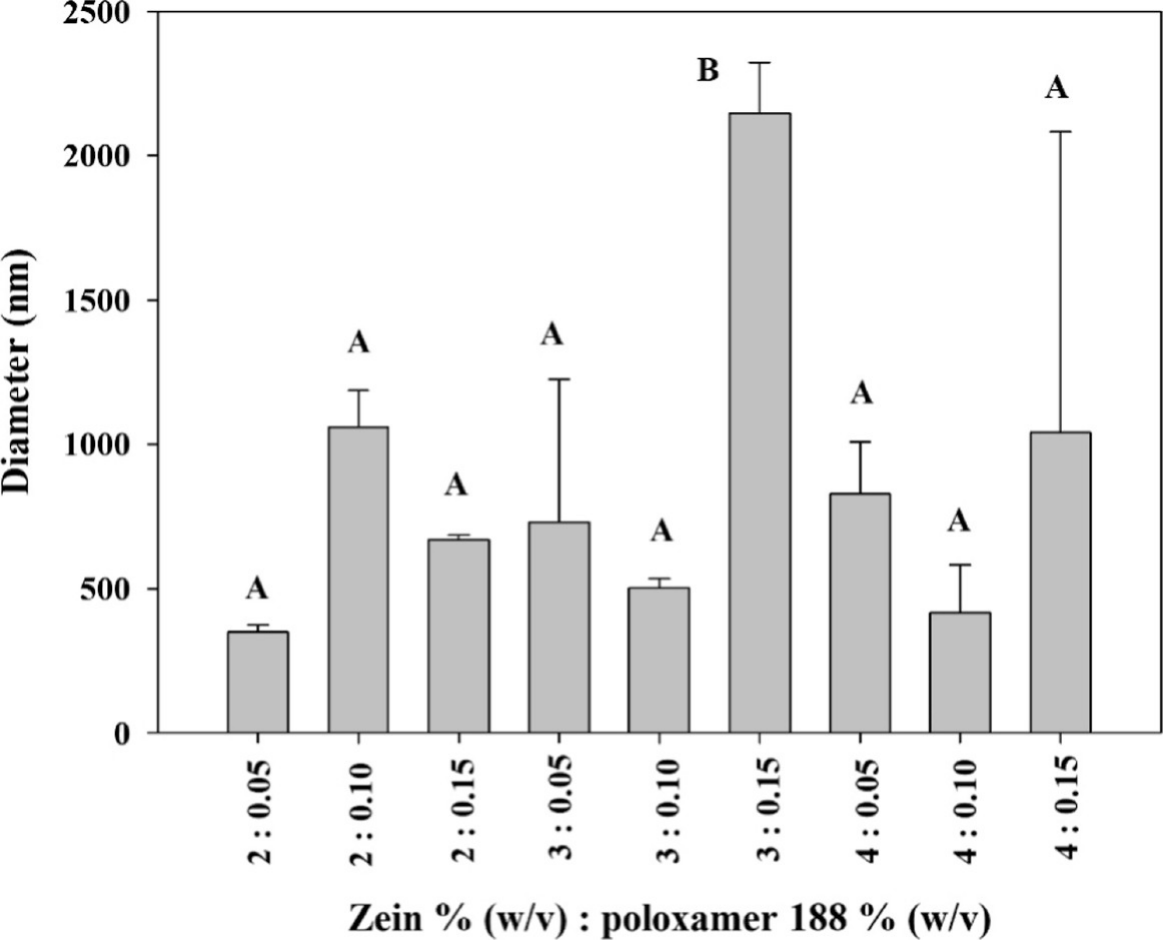
Effect of zein concentration and poloxamer 188 on the zein particle size without phosphate.

**Table 3:** Means of Particle Size, Polydispersity Index, and Z-Potential of the Zein Nanoparticles^a^

zein % (w/v)	poloxamer 188% (w/v)	size (nm)	polydispersity index	z-potential (mV)
2	0.05	348 ± 23^a^	0.6 ± 0.1^b^	6.4 ± 0.5^a^
2	0.1	1059 ± 216^b^	0.2 ± 0.1^a^	20.2 ± 0.9^c^
2	0.15	667 ± 17 ^ab^	0.4 ± 0.1^b^	16.6 ± 3.1^b^
3	0.05	728 ± 496 ^ab^	0.9 ± 0.1^c^	31.2 ± 1.2^f^
3	0.1	501 ± 85^a^	0.4 ± 0.2 ^ab^	27.9 ± 1.1^e^
3	0.15	2146 ± 177^c^	0.4 ± 0.1^b^	20.3 ± 1.7^c^
4	0.05	829 ± 348 ^ab^	0.9 ± 0.2^c^	22.7 ± 1.0 ^cd^
4	0.1	415 ± 174^a^	0.9 ± 0.1^c^	24.8 ± 1.0^d^
4	0.15	552 ± 355^a^	0.9 ± 0.7^c^	5.1 ± 0.9^a^

a The values correspond to the means ± standard deviation of six measurements, with different letters in each column for each sample, if they are significantly different (*p* < 0.05) with 95% confidence.

**Table 4:** Means of Particle Size, Polydispersity Index, and Z-Potential of Zein Nanoparticles Loaded with Monobasic Phosphate Monohydrate

zein % (w/v)	poloxamer 188% (w/v)	Ca(H_2_PO_4_)_2_·H_2_O % (w/v)	size (nm)	polydispersity index	z-potential (mV)
2	0.05	1	1566 ± 27	0.4 ± 0.1	12.8 ± 0.7
2	0.05	2	1880 ± 226	0.5 ± 0.1	17.7 ± 0.8
2	0.05	3	3568 ± 1466	0.8 ± 0.2	0.8 ± 0.4
2	0.1	1	3037 ± 260	0.4 ± 0.1	29.5 ± 2.6
2	0.1	2	2378 ± 165	0.4 ± 0.1	32.4 ± 14.5
2	0.1	3	3070 ± 835	0.7 ± 0.2	18.7 ± 0.9
2	0.15	1	1334 ± 102	0.4 ± 0.1	43.1 ± 5.9
2	0.15	2	3538 ± 750	0.5 ± 0.1	33.6 ± 4.1
2	0.15	3	3063 ± 1018	0.6 ± 0.2	32.05 ± 1.9
3	0.05	1	1540 ± 103	0.4 ± 0.1	21.1 ± 8.6
3	0.05	2	1587 ± 422	0.7 ± 0.3	54.7 ± 4.6
3	0.05	3	1123 ± 325	0.7 ± 0.3	50.4 ± 4.0
3	0.1	1	1525 ± 372	0.9 ± 0.03	35.7 ± 7.5
3	0.1	2	2013 ± 862	0.9 ± 0.1	42.4 ± 2.9
3	0.1	3	2881 ± 1783	0.8 ± 0.2	43.5 ± 6.3
3	0.15	1	367 ± 16	0.3 ± 0.1	79.8 ± 11.6
3	0.15	2	326 ± 15	0.3 ± 0.1	178.8 ± 9.9
3	0.15	3	375 ± 11	0.2 ± 0.1	125.8 ± 6.5
4	0.05	1	2478 ± 88	0.3 ± 0.04	46.8 ± 3.4
4	0.05	2	1907 ± 464	0.8 ± 0.3	50.5 ± 4.5
4	0.05	3	1835 ± 375	0.6 ± 0.2	43.6 ± 3.5
4	0.1	1	3069 ± 382	0.3 ± 0.2	44.03 ± 2.2
4	0.1	2	1958 ± 84	0.4 ± 0.1	21.9 ± 3.5
4	0.1	3	2324 ± 394	0.5 ± 0.2	33.0 ± 2.01
4	0.15	1	1321 ± 343	0.7 ± 0.2	44.6 ± 5
4	0.15	2	2356 ± 1141	0.9 ± 0.04	47.4 ± 2.8
4	0.15	3	2315 ± 433	0.7 ± 0.1	32.6 ± 7.0

An important parameter when considering the size of the synthesized nanoparticles is the surface area. The smaller the size, the greater the surface area. In the case of protein nanoparticles, this means that the protein structure remains the same as the native one and increases its intrinsic activity, while its size decreases. A smaller size indicates a larger contact surface; therefore, the unique properties of this nanoscale material can be better exploited.

Zein-phosphorus nanoparticles freeze-dried. Significant differences were observed regarding the effect of the concentration of zein (*F*(2) = 26.5, *p* < 0.0001) and poloxamer (*F*(2) = 8.85, *p* = 0.0002), but not in the concentration of monobasic calcium phosphate (Ca­(H_2_PO_4_)_2_·H_2_O) (*F*(2) = 2.41, *p* = 0.093) on the size of the nanoparticles formed (Tables 3 and 4). The use of poloxamer 188 can lower the viscosity of nanoparticles in suspension.^85^ Considering that the diffusion medium is water, this could reduce resistance to the release of phosphorus, which can be utilized by plants.

The 3% (w/v) concentration of zein was significantly smaller (1304 nm) compared to the 2% (w/v) (2586 nm) and 4% (w/v) (2174 nm) concentrations; the last two did not present significant differences between them. While in the concentrations of poloxamer used, a significant increase in size was observed in the particles with a concentration of 0.1% (w/v) (2461 nm), unlike the concentrations of 0.15 and 0.05% (w/v) that presented average sizes of 1640 and 1942 nm, respectively. Finally, the phosphorus concentrations used were not significantly different in any of their concentrations; therefore, no effect was observed on them.

The average size of the particles formed exceeded 1000 nm in 87.7% of the cases, which is why they are considered microparticles. However, the results for the purposes of this research may be useful, since it has been previously reported that nanoparticles that are too small (>90 nm) and mainly made of metallic compounds have bioaccumulation effects and can cause oxidative stress in plants.^68^ In addition, nanoparticles with a size between 20 and 90 nm can inhibit enzymatic activity and nitrification in beneficial bacteria soil.^68^ Other authors,^50^ consider that, at appropriate dosage, nanofertilizers made from metals considered micronutrients for plants can serve as nutritional mediators and promote stress tolerance. Although, the size of the particles formed by adding phosphorus increased in most cases, except for those formed with zein at 3% (w/v) and 0.15% (w/v) of poloxamer, in which their average size decreased with respect to their concentrations without adding phosphorus, these nanoparticles have a size between 325 to 375 nm and polydispersity index on zein nanoparticles with and without phosphorus. The polydispersity index presented on lyophilized zein nanoparticles in concentrations of 2, 3, and 4% (w/v) of zein was 0.4, 0.56, and 0.89, respectively. The particles formed from 2 and 3% (w/v) of zein had significant differences with the concentration of 4% (w/v) but not between them. Therefore, it was observed that increasing the zein concentration also increases the polydispersity index. That is, there is greater variability in the particle size. Sun et al.,^90^ attribute this characteristic to the mechanism by which nanoparticles or microparticles are formed. In the case of nanoparticles, their union is favored by Brownian motion and attractive surface interactions. On the other hand, when the concentration increases under the same conditions, the coagulation mechanism changes from charge neutralization to sweep flocculation, and the particles become destabilized and restabilized constantly.

Regarding the effect of the concentration of poloxamer 188 (w/v) used, the concentrations of 0.1 and 0.15% (w/v) were significantly different (*F*(2) = 5.9, *p* = 0.005) from the concentration of 0.05% (p/v), this being the one that presented the highest polydispersity index (0.78). Concentrations of 0.1 and 0.15% (w/v) poloxamer presented polydispersity indexes of 0.49 and 0.56, respectively, which presented significant differences between them. The above is attributed to the particle size, which is influenced by the surfactant nature and concentration.^91^ The poloxamer 188 can produce highly organized nanostructures, with chemically reactive and functionalized surfaces.^92^ This could be explained due to the hydrophobic part of poloxamer 188 interacting with the surface of zein nanoparticles through hydrogen bonds, which causes the hydrophilic part of the molecule to be exposed, and increases its polarity^80^ (Figure 11). The hydrophilic part of the poloxamer (polyethylene glycol) provides steric hindrance that decreases the probability of dominant hydrophobic interactions, favors colloidal stability, and decreases aggregation.^76^ Poloxamer 188 facilitates an equilibrium in which hydrophilic interactions (provided by the polyethylene glycol chains) can balance out the hydrophobic interactions of the particle and help to maintain a stable dispersion in aqueous media.^93^ For this reason, the surfactant is necessary to preserve the nanoparticle suspensions to avoid agglomeration over long periods of time.^70^ Therefore, the decrease in the concentration of poloxamer 188 could cause the formation of nanoparticles to be more unstable and their polydispersity to increase. Göke et al.,^94^ used poloxamer 188 to prepare nanoparticles under thermal treatment and found an increase in particle size and a decrease in particle size distribution width at the same time. In this experiment, similar results were observed when increasing the concentration of poloxamer 188, since at a higher concentration the size increased but the polydispersity index also decreased; however, the mechanism by which this happens is not yet well elucidated.

Regarding the polydispersity index of phosphorus freeze-dried zein nanoparticles, the concentration of zein used had no effect on it (*F*(2) = 0.83, *p* = 0.44), nor did the concentration of poloxamer (*F*(2) = 1.62, *p* = 0.20), but in the concentration of monobasic calcium phosphate (*F*(2) = 7.4, *p* = 0.0008) (Table 4). The nanoparticles that used the lowest concentration (1% w/v) presented a significantly lower value in the polydispersity index with respect to the other concentrations of 2 and 3% (w/v) used. Monobasic calcium phosphate is a complex salt; on the other hand, studios for the elaboration of zein nanoparticles, salts are utilized like ionic surfactants.^95^ This interaction can be initiated by the electrostatic force between the charged headgroups of surfactants and the oppositely charged amino acid residues of proteins.^96^ There are different theories that could explain the differences between the polydispersity index and the concentration. (1) Nucleation and growth theory: the nucleation process occurs when zein molecules aggregate through hydrogen bonds and electrostatic forces, which grow as more molecules are incorporated. At higher zein concentrations, nucleation increases and can generate particles of different sizes; therefore, the presence of high concentrations of phosphate can increase the size by interacting with the carboxyl groups of the zein and cause variations in its size.^77,97^ (2) Derjaguin–Landau–Verwey–Overbeek (DLVO) theory: this theory considers the sum of the van der Waals attraction forces and electrostatic repulsion in colloidal solutions. As is the case for zein nanoparticles suspended in 70% (v/v) ethanol at higher concentrations, the proximity between nanoparticles increases and can intensify the interactions and reduce the effectiveness of the stabilizing repulsive forces. Said behavior favors particle aggregation and contributes to greater polydispersity.^96^ (3) Effect of the viscosity of the medium: a significant increase in concentration can increase the viscosity of the medium and affect the dynamics of particle formation. Therefore, higher viscosity makes it difficult for zein molecules to diffuse into the medium and generates a heterogeneous mixture that results in the formation of particles of different sizes.^77^

Polydispersity index is important, due to its relationship with the molecular weight of amorphous polymers, and since it influences certain physical characteristics of the polymer, such as melt viscosity, tensile strength, resistance to impact or toughness, and resistance to heat and corrosives.^98^ Another authors,^99,100^ reported that stability and encapsulation efficiency, among other properties of nanoparticles, were determined by particle size.

Z-potential of zein nanoparticles with and without phosphorus by nanoprecipitation. Significant differences were observed on lyophilized zein nanoparticles regarding the effect of the concentration of zein (*F*(2) = 14.32, *p* < 0.0001) and poloxamer 188 (*F*(2) = 7.34, *p* = 0.0016) on the z-potential (Table 3). Z-potential is related to the surface charge of the zein nanoparticle, and this is mainly due to the charge presented by its amino acids according to pH solution. Concentrations of 2 and 4% (w/v) of zein particles presented means of 14.39 and 18.22 mV, respectively, and were significantly lower than those presented with a concentration of 3% (w/v) of zein (26.34 mV). The fact that there is no relationship between the concentration of zein and the z-potential suggests that the zein nanoparticles are sterically stabilized,^101^ that is, coated with a polymer, which in this case could be poloxamer 188. While the influence of the concentration of poloxamer 188 (w/v) was observed, the highest z-potential mean values were observed at the concentration of 0.1% (w/v) reaching 24.19 mV. No significant difference was observed in z-potential values between 0.05% (w/v) (20.11 mV) and 0.1%. However, significant differences in z-potential values were noted for 0.15% (w/v) (14.45 mV). Podaralla and Perumal^97^ found that the surfactants and therefore electrostatic interactions may not play a predominant role in the z-potential of nanoparticles. Based on these results, concentrations with 0.1% (w/v) poloxamer 188 were the most stable suspensions of nanoparticles (Table 2).

The value of the z-potential is an indication of the stability of the particles in suspension. Strongly positive (+30 mV) or strongly negative (−30 mV) values will tend to repel each other due to the equality of charges and therefore will not add to each other, unlike values close to zero, where the suspension is mixed and considered unstable and the particles will end up aggregating and precipitating.^102^ However, in Figure 5 it is possible to observe the formation of apparently stable nanoparticles even when their z potential values indicate lower than 30 mV. In the case of nanoparticles composed of proteins, it is possible that they show stability even if their z potential values are below those mentioned (⟨30 y⟩ −30 mV). This may be due to mechanisms such as hydration, the steric effect, and secondary repulsive interactions. When proteins form nanoparticles, they are surrounded by a layer of water that causes steric hindrance and prevent forces such as van der Waals from inducing aggregation.^103^ Dipolar interactions or the three-dimensional conformation of the protein can generate additional repulsive forces, which contribute to colloidal stability.^104^ Therefore, given that mechanisms such as hydration and other repulsive forces can contribute to stability, nanoparticles made from proteins do not depend exclusively on the z potential for their stability.

Significant differences were obtained on z-potential values on zein-phosphorus freeze-dried nanoparticles with respect to the concentration of zein used (*F*(2) = 31.93, *p* < 0.0001) and poloxamer (*F*(2) = 23.49, *p* < 0.0001) (Table 3). However, no significant difference was found with respect to the concentrations of monobasic calcium phosphate (*F*(2) = 2.2, *p* = 0.1147) used. Therefore, the concentration of monobasic phosphate does not affect the stability of the particle according to the results of the z-potential. From the previous analysis, the most stable samples were taken to perform SEM and observe their morphology (Table 2).

It is possible to observe that the nanoparticles in Table 2 present high levels of z potential. This indicates good nanoparticle stability that prevents their aggregation. However, it is important to discuss the possible effects that these z potential values could have on plants.^105^ Plant cells are negatively charged^106^ and will be attracted to positively charged particles, in turn, this can increase their affinity and facilitate their translocation.^107^ In the case of nanoparticles between 50 and 200 nm, translocation to plant cells occurs mainly by the apoplastic pathway,^51^ some authors^108^ suggest that a size greater than 140 nm prevents translocation through the plant cell and accumulation in roots. Therefore, the above suggests that nanoparticles with added phosphate of a size around 350 nm will not tend to accumulate in the roots but will be attracted to plant cells due to their positive surface charge. In addition, due to their size, they will not be able to translocate through the plant, as in the case of metallic nanoparticles, which helps to avoid toxicity.

The dispersion speed through soil can contribute to or decrease the leaching of nanofertilizers. Liu & Lal^109^ found that hydroxyapatite nanoparticles, with an average size of 15.6 nm, as a source of phosphorus were distributed more slowly in soil, which helped to reduce eutrophication. Provided that the nanoparticles in this research are larger, their diffusion through the soil is expected to be slower. In addition, it is important to add that, since they are made from a biodegradable and nontoxic biopolymer such as zein, the nanoparticles will be degraded upon contact with environmental factors.

### Rheology

In Figure 7, the apparent viscosity and shear stress were analyzed for zein nanoparticles at 3% (w/v) with 0.15% (w/v) of poloxamer 188 and zein nanoparticles of the same concentration, but with 1 and 2% (w/v) Ca­(H_2_PO_4_)_2_·H_2_O at shear rates of 1 to 300 s^–1^. It was found that zein nanoparticles have a non-Newtonian dilatant behavior since they have an *n* value greater than 1 (Table 5). This agrees with the nature of the sample because it is a high-concentration solution that has particles in the suspension. These particles, when at rest, have low viscosity, but when the shear stress increases, the liquid in which they are suspended is displaced, which causes friction between the particles and increases their viscosity.^110^ It is also possible to observe an instability of viscosity with respect to shear speed, and this is mainly because the solutions are not completely dissolved because they are suspensions.^111^

**Figure 7:**
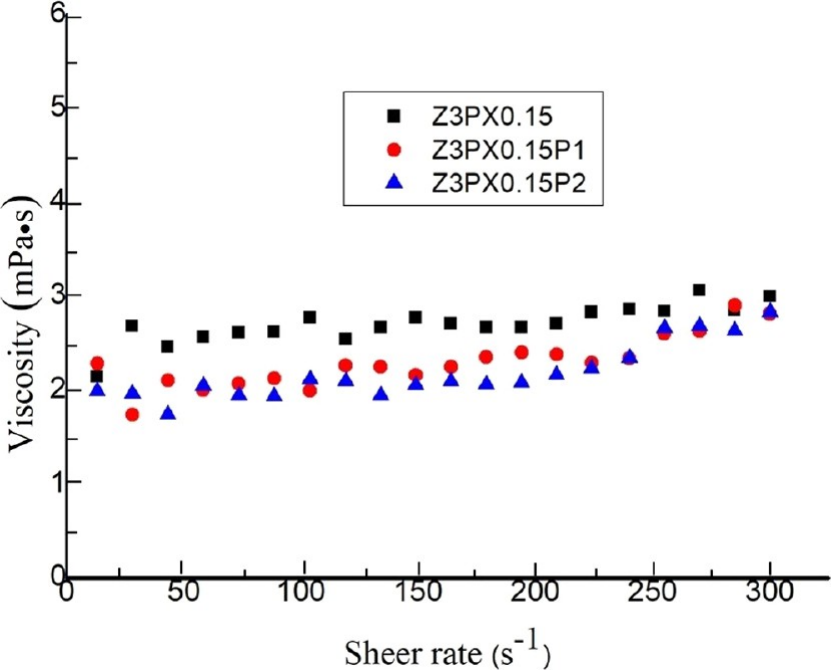
Dependence of the viscosity of nanoparticle solutions on the shear rate. Zein nanoparticles are represented by the black square (Z3PX0.15), zein nanoparticles with 1% (w/v) phosphate are represented by red circle (Z3PX0.15P1), and these same nanoparticles with 2% (w/v) phosphate are represented by blue triangle (Z3PX0.15P2). Zein 3% (w/v) = Z3, poloxamer 188 0.15% (w/v) = PX0.15, phosphate 1% (w/v) = P1, and phosphate 2% (w/v) = P2.

**Table 5:** Values Obtained from the Power Law Variables from the Analyzed Samples^a^

sample	*K*_viscosity_	*n*_viscosity_	*R* ^2^_viscosity_
zein nanoparticle	0.002	0.06	0.52
zein nanoparticle P1	0.002	0.06	0.25
zein nanoparticle P2	0.0009	0.15	0.61

sample	*K*_sheer rate_	*n*_sheer rate_	*R* ^2^_sheer rate_
zein nanoparticle	0.002	1.06	0.99
zein nanoparticle P1	0.002	1.06	0.99
zein nanoparticle P2	0.009	1.15	0.98

a P1 = 1% Ca­(H_2_PO_4_)_2_·H_2_O (w/v); P2 = 2% Ca­(H_2_PO_4_)_2_·H_2_O (w/v).

Figure 7 shows the behavior of the zein nanoparticles and zein-phosphate nanoparticles. It is possible to observe that zein-surfactant nanoparticles are more viscous than zein-phosphate nanoparticles. The above can be due to the networks formed when interacting with ethanol and phosphate, since it can act as a plasticizer.^112^ Therefore, the presence of both compounds in ethanol could act as a lubricant, promoting molecular movement and reducing friction between the chains.^113^ For this reason, zein-phosphate nanoparticles are less viscous than zein nanoparticles alone.

Total zein (which contains the different types of zein: α, β, and δ) can form networks when in contact with a plasticizer that increases its viscoelasticity, such as ethanol and poloxamer 188.^112^ Besides this, the Ca^2+^ ion present in the dissociation of monobasic calcium phosphate monohydrate may contribute to this. The possibility of forming zein networks is related to the presence of a high amount of α-zein, the most abundant in hydrophobic interactions, when hydrophobicity decreases, as well as the possibility of forming networks due to the protein–protein interaction which decreases and it is not possible to form aggregates.^114^ Another authors,^115–117^ attribute the formation of zein networks to a decrease in α-helices in the secondary structure and an increase in the number of β-sheets, which maintains a positive correlation with its viscoelasticity. On the other hand, Zhang et al.,^112^ found that the structure of the zein, when going from a solid particle to a viscoelastic network, tended to decrease the order of its secondary structures. It is possible that the lyophilization process of the sample could impact on the protein and its stability.^118^

### Fourier Transform Infrared Spectroscopy with Attenuated Reflectance (FTIR-ATR)

The FTIR spectra were analyzed for each component of the nanoparticles separately (zein, poloxamer 188, and monobasic calcium phosphate monohydrate) (Figure S1). Subsequently, the FTIR spectra of the nanoparticles with and without phosphate were analyzed (Figure 8). Infrared spectroscopy allows for the determination of the formation of new bonds between the compounds involved.^119^ Infrared spectrum of phosphorus zein nanoparticles. In the case of nanoparticles loaded with phosphorus, it is possible to observe changes in the spectrum with respect to unloaded nanoparticles. The most significant differences are found in the band between 955 and 976 cm^–1^ corresponds to the phosphate group (PO_4_^3–^),^120^ and appears in the nanoparticles with 1 and 2% w/v of phosphate respectively, which intensifies with increasing concentration. This shift toward lower frequencies is due to the increase in intermolecular interactions that we can attribute in this case to hydrogen bonds between the oxygen of the phosphate and the hydrogen of the aliphatic chain of the amino acids, and the hydrogen of the phosphate and the oxygen of the glutamic acid^80^ (Figure 11). Whereas the most characteristic band of poloxamer 188 appears at 1106 cm^–1^ due to C–O stretching on zein nanoparticles.^121^ When phosphate is added, it is possible to see a shift toward lower wavelengths (Figure 8), which indicates its interaction with the phosphate group through hydrogen bonds (Figure 11). Furthermore, it is possible to appreciate an increase in the band of –OH, due to the stretching of the structural –OH group and of the absorbed water^120^ (Figure 8). With the above, experiments were carried out; it was possible to obtain zein nanoparticles with phosphorus trapped in their structure.

**Figure 8:**
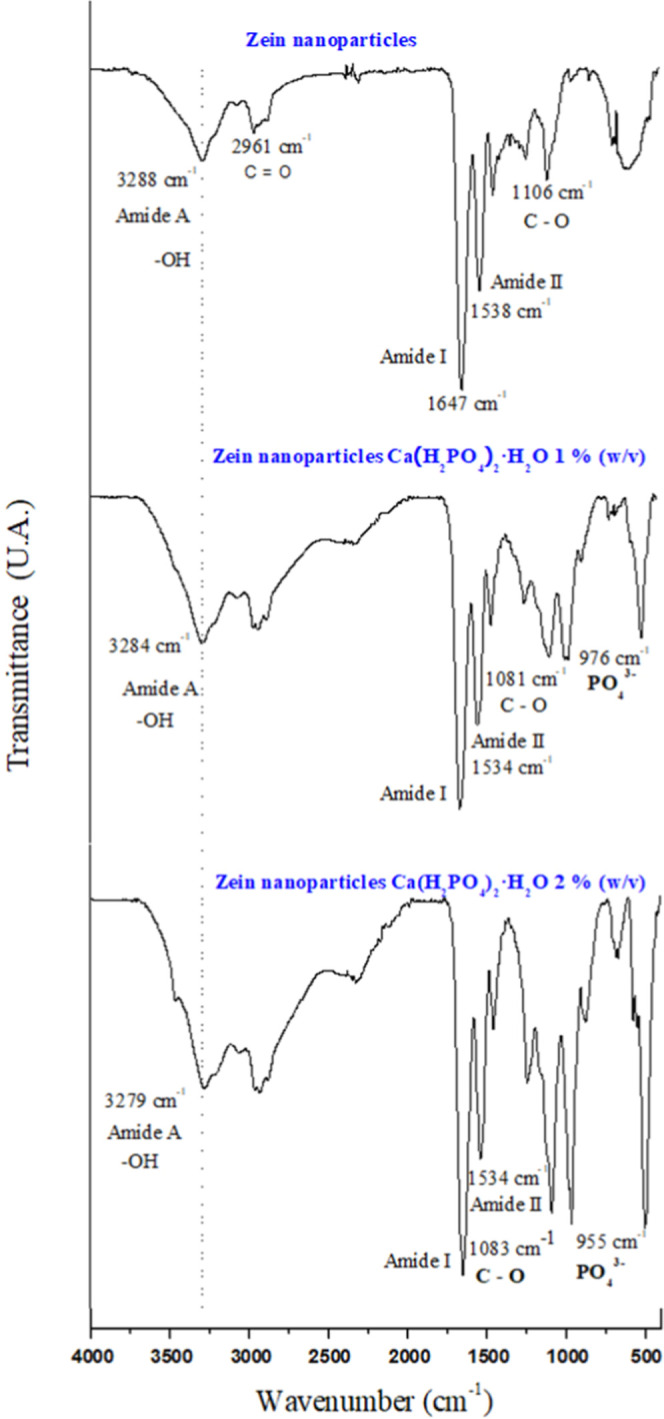
Infrared spectrum of 3% (w/v) zein nanoparticles, 0.15% (w/v) poloxamer, and 1% (w/v) and 2% Ca­(H_2_PO_4_)·H_2_O compared to nanoparticles of the same concentration of zein without trapped phosphorus.

Deconvolution

Lyophilized samples of zein nanoparticles were analyzed by FTIR deconvolution analysis. It is possible to determine from amide I (1750–1570 cm^–1^), the predominant secondary structure of the protein. This is attributed to C–O stretching of the amide group, coupled with bending of the N–H bond.^122^ The distribution of the secondary structure of amide I is as follows: α-helix (1650–1660 cm^–1^); β sheets (1620–1640 cm^–1^); β turn (1660–1675 cm^–1^) and high frequency of β sheets (1675–1700 cm^–1^).^123^ Regarding the secondary structure, the zein is composed mainly of α-helices; however, β-sheets may also be present in the structure in a lesser proportion. For the deconvolution analysis of the zein concentration at 3% (w/v) with 0.15% (w/v) poloxamer 188 and 1% (w/v) Ca­(H_2_PO_4_)_2_·H_2_O, a 91.3% relationship was observed with the α-helix structure corresponding to the wavenumber 1656 ± 2 cm^–1^.^124^ Regarding the deconvolution analysis of the zein at 3% (w/v) with 0.15% (w/v) poloxamer 188 and 2% (w/v) Ca­(H_2_PO_4_)_2_·H_2_O, a 97.4% relationship was observed with the β-sheet structure corresponding to the wavenumber 1627 ± 2,^124^ which can be related to the model of Wang and Padua.^5^ Erickson et al.,^125^ reported that when commercial zein self-assembles into a viscoelastic network, the β sheet increases and the α helix decreases; that is, the proportion of α zein (which is the most abundant) interacts mainly through noncovalent interactions that are responsible for forming the viscoelastic network.^126^ From the information presented above, it is possible to see that the secondary structure of the nanoparticle is not affected by lyophilization.

The amide II region is related to N–H bending and C–N stretching; it is more related to the viscoelastic capacity of the protein and not so much to its secondary structure.^115^ This is mainly because the amide II region is more sensitive to protein–solvent interactions, making it more prone to hydration and less reliable for secondary structure determination.^127^ Therefore, only amide I was considered for the determination of the secondary structure of the zein nanoparticles formed. The secondary conformation of the zein can change depending on the solvent used, the technique to which the protein is subjected, and whether it is found pure in solution or in combination.^128^ However, Forato et al.,^129^ concluded in their work that extraction and solubilization with ethanol did not affect the proportion of zein secondary structures.

The selected concentrations for FTIR analysis and their deconvolution were selected that present high z-potential (zein 3% w/v, poloxamer 0.15% w/v) and (Ca­(H_2_PO_4_)_2_·H_2_O 1% and 2% w/v) because these samples presented the highest stability. Likewise, it was possible to appreciate the characteristic band of the phosphate group in the nanoparticles with trapped Ca­(H_2_PO_4_)_2_·H_2_O, unlike that of the uncharged nanoparticles. This is key to being able to use these nanoparticles as a potential prolonged-release fertilizer.

### Differential Scanning Calorimetry

The behavior with respect to phase changes in the glass transition temperature and melting points of zein (*T*_g_ 87 °C), poloxamer 188 (*T*_m_ 58 °C), monobasic calcium phosphate monohydrate (*T*_g_ 173 °C), zein nanoparticles (*T*_g_ 88 °C), and zein-Ca­(H_2_PO_4_)_2_·H_2_O nanoparticles with 1 (*T*_g_ 110 °C) and 2% (121 °C) were evaluated using this technique (Figures 9 and 10). This technique determines the thermal properties of a material as a function of temperature, such as the glass transition temperature (*T*_g_), melting temperature (*T*_m_), and crystallization of the material.^130^

**Figure 9:**
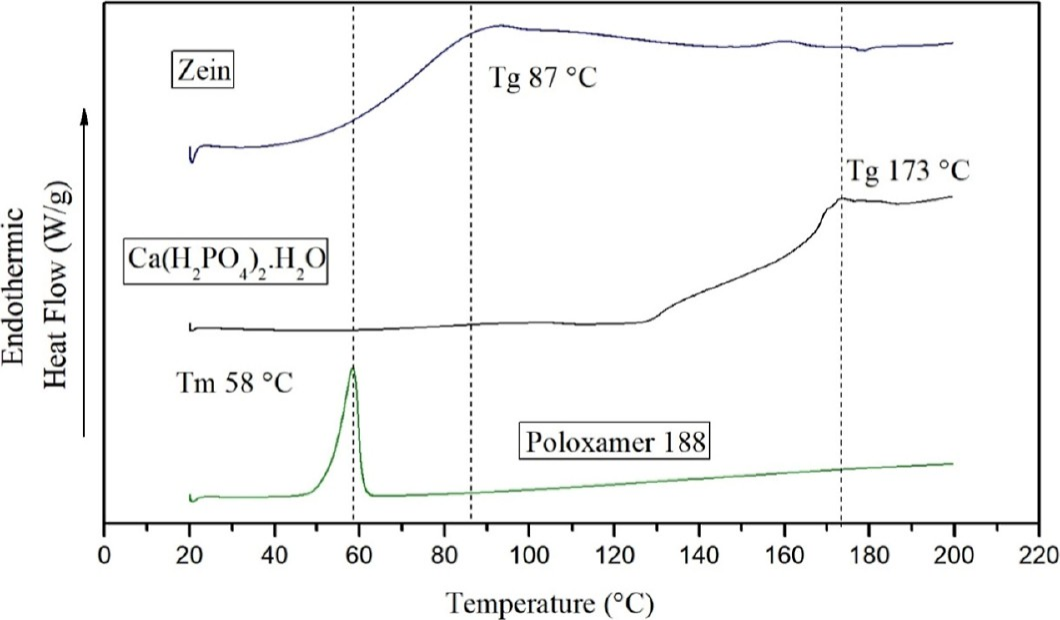
DSC analysis of nanoparticle components zein, monobasic calcium phosphate monohydrate, and poloxamer 188.

**Figure 10:**
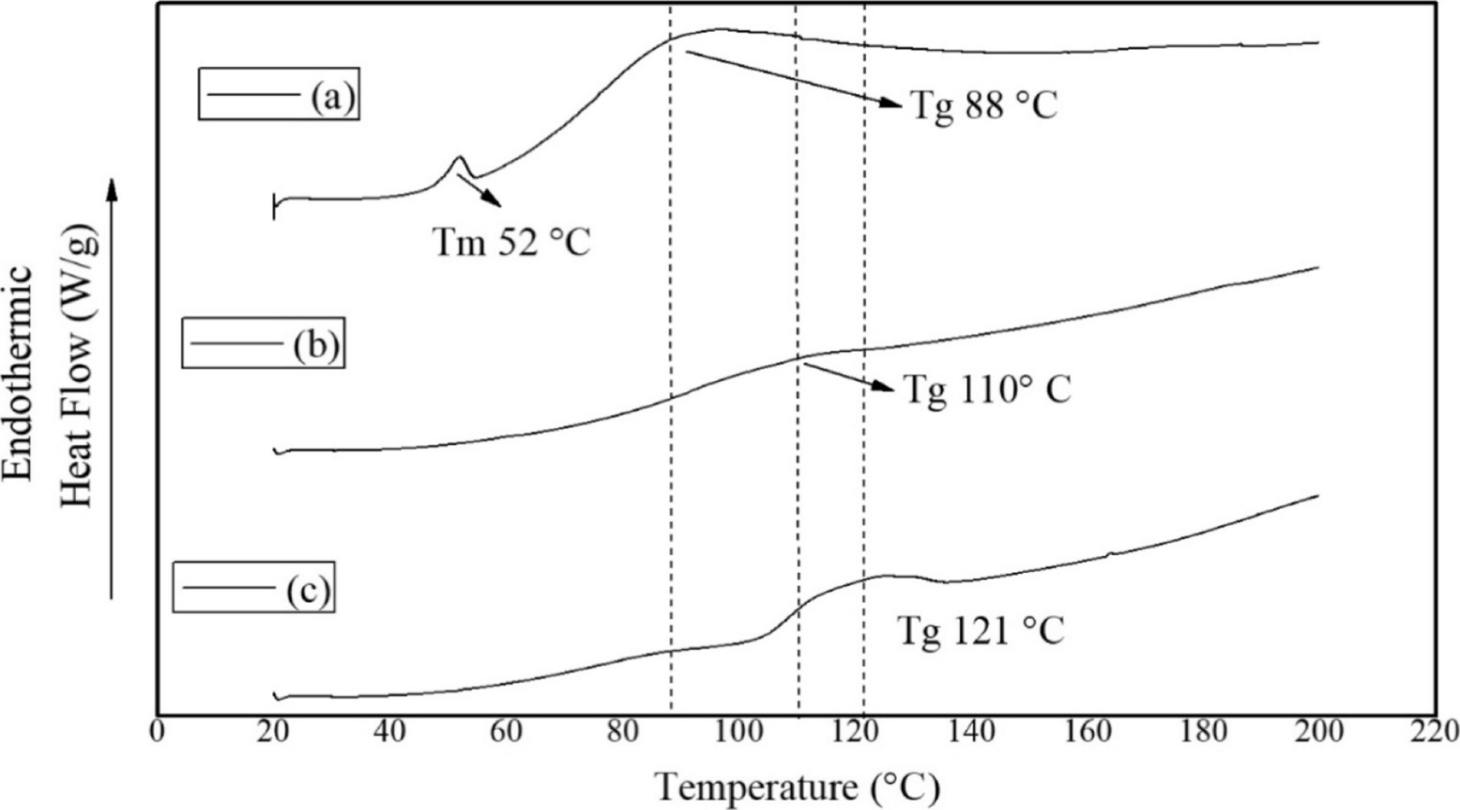
DSC thermograms of nanoparticles with zein concentration 3% (w/v), poloxamer 188 0.15% (w/v), without phosphorus (a), and concentrations of 1% (b), and 2% (c) w/v of Ca­(H_2_PO_4_)_2_·H_2_O.

In general, zein shows a thermal transition at 87 °C (Figure 9) corresponding to the glass transition, because it is an amorphous polymer.^131^ Zhang et al.,^112^ report that for alpha zein with a humidity percentage close to 12%, a *T*_g_ like that reported in this work would be obtained. The glass transition reported in the literature could vary depending on the purity of the zein used, its composition, the extraction method, and the conditions of the determination method (such as heating rate).^132^ Furthermore, a decrease in *T*_g_ is related to the presence of water.^126^ Therefore, the more hydrated the zein is, the more its *T*_g_ will decrease. This may directly affect its ability to form cross-links or networks, since zein can form networks only above the temperature of its glass transition.^115,133^

Poloxamer 188 can be decomposed on formic acid, acetic acid, formaldehyde, acetaldehyde, and low-molecular-weight polymers by autoxidation of the chains.^134^ The concentration used in this experimental study was 0.15% (w/v). Concentrations of the surfactant (poloxamer 188) should be used in small amounts to avoid the risk of gelation unless otherwise desired.^135^ It is possible to observe the melting peak at 52 °C of poloxamer 188 in the nanoparticles without phosphate present ((a), Figure 10); this corresponds to the unbound poloxamer 188 of the formulation; however, it disappears once phosphate (Ca­(H_2_PO_4_)_2_·H_2_O) has been added. This could be due to the interaction of the molecule with the phosphate group of Ca­(H_2_PO_4_)_2_·H_2_O and zein through the formation of hydrogen bonds between the OH groups. The pH of the nanoparticles is close to 7, therefore the present ionizable forms of the Ca­(H_2_PO_4_)_2_·H_2_O salt, according to its titration curve, are [H_2_PO_4_^–^] = [H_2_PO_4_^2–^]. Being polar molecules, they could form hydrogen bonds, as illustrated in Figure 11.

**Figure 11:**
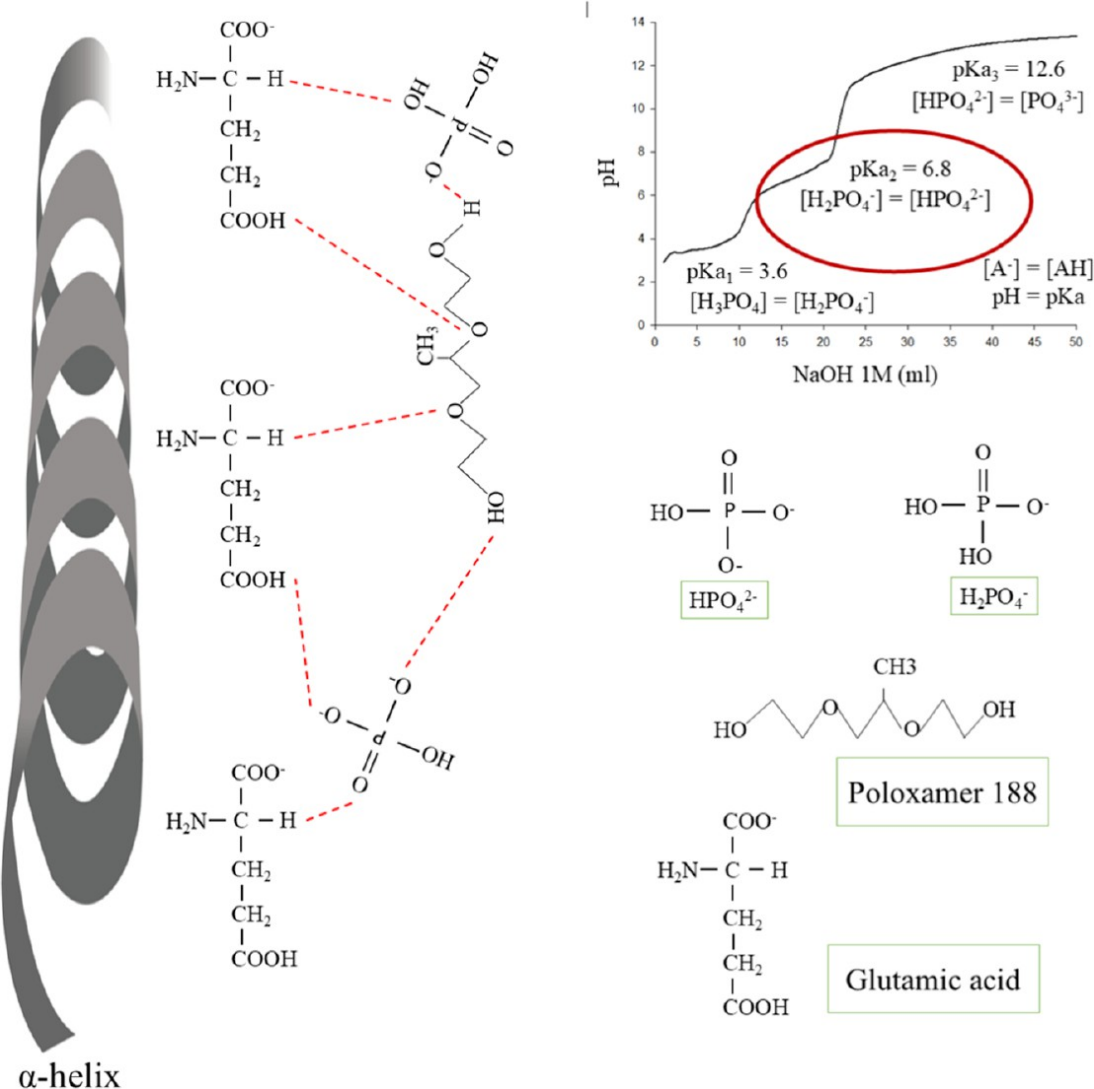
Interactions of hydrogen bounds between ionizable forms of Ca­(H_2_PO_4_)_2_·H_2_O, glutamic acid of zein, and poloxamer 188.

Finally, in the case of phosphate (Ca­(H_2_PO_4_)_2_·H_2_O), a wide band is observed where the highest point of the transition is 173 °C corresponding to its *T*_g_ (Figure 10), since it is an amorphous material,^136^ and the loss of the water molecule.^137^ It is possible to observe that at the time it was added to the zein nanoparticles in concentrations of 1 and 2% (w/v), the melting point of the zein that appears at 88 °C in the nanoparticles without phosphate was shifted to 110 °C in the concentration at 1% (w/v) and to 121 °C in the case of 2% (w/v) (Figure 10). Furthermore, an increase in the curve (c) nanoparticles corresponding to the increase in the phosphate concentration is observed (Figure 10). In the case of nanoparticles without added phosphate (a, Figure 10), these present a melting point that corresponds to poloxamer 188 that did not bind to the zein nanoparticles and a band where the highest peak is 88 °C, corresponding to the glass transition. The shift in the glass transition is due to the increase in the side chains of the zein, corresponding to the formation of hydrogen bonds between the zein, poloxamer 188, and phosphate. The polar regions of the latter increase the polarity of the material, which also contributes to the displacement of the glass transition.

The glass transition and melting point of the material indicate the type of ordering of the nanoparticle chains. Zein nanoparticles with and without trapped phosphate are amorphous. The presence and higher concentration of phosphate (Ca­(H_2_PO_4_)_2_·H_2_O) shifted the glass transition of the zein nanoparticles, indicating their interaction. With the above, it is possible to clarify that the phosphate is trapped in the nanoparticle zein and can change its thermal behavior, in addition to making it more stable.

### Prolonged Release of Ca­(H_2_PO_4_)_2_·H_2_O from Zein Nanoparticles

Figure 12 presents the results of the release of Ca­(H_2_PO_4_)_2_·H_2_O from the zein nanoparticles. Zein nanoparticles with 2% (w/v) of phosphate released around 20% of the phosphate during the first 600 min. The exponential release during the first 100 min is due to the phosphate found on the surface of the zein nanoparticles, which is in contact with the aqueous medium and released quickly. This phenomenon is called “exploiting”. The percentage of release after 600 min remained relatively constant, so it is assumed that the system reaches equilibrium. The difference between Ca­(H_2_PO_4_)_2_·H_2_O as a conventional fertilizer and zein nanoparticles with trapped Ca­(H_2_PO_4_)_2_·H_2_O is the release time. The amount of phosphate released at 600 min corresponds to 0.038 g of 0.19 g added to the system that were released gradually, while the same amount of phosphate added to the system is immediately solubilized (as it is a salt). Since it is more likely that a significant percentage of the fertilizer used conventionally is lost through leaching than with a slow-release fertilizer, this would have an impact when used in the field.

**Figure 12:**
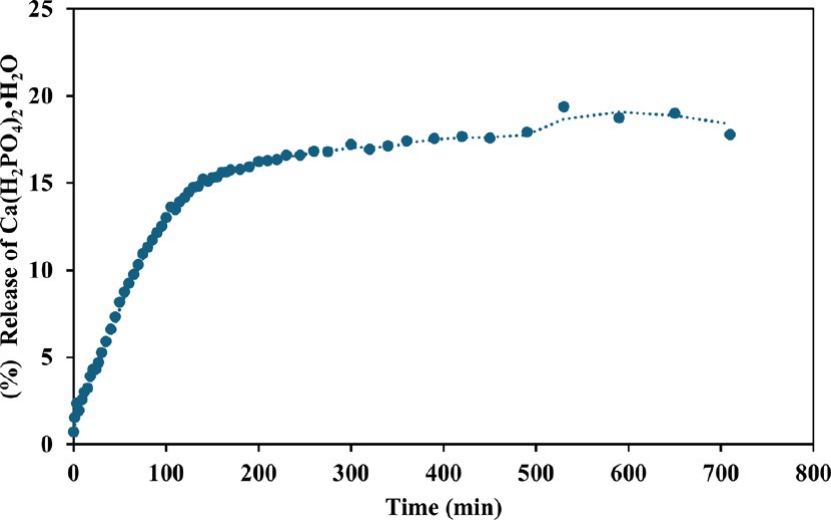
Prolonged release of Ca­(H_2_PO_4_)_2_·H_2_O 2% (p/v) from zein nanoparticles on water.

Other authors report different release percentages for systems based on zein matrixes. Alinaqi et al.,^138^ reported a release close to 45% of clove essential oil during the first 10 h and around 80% after 120 h. Chen et al.^139^ reported release percentages between 50 and 85% of casein and curcumin in zein nanoparticles under the effect of different stabilizers. Finally, Lima et al.^140^ reported the effect of pH on the release of mesalazine from zein nanoparticles. For such, it is possible to improve release percentage by modifying some variables, such as pH, used stabilizer, or test length. Changes in the pH of the system could have a greater influence on the percentage of released phosphate. It should be noted that it is necessary to maintain a pH close to that of the soil when the release kinetics. By acidification of the medium, below the isoelectric point of zein, the surface charge of zein would change because the amino groups would gain a proton, and the negatively charged carboxyl groups would be neutralized. This would result in a reduction in the electrostatic interaction between zein and calcium phosphate, which could cause particle aggregation.^141^ In the case of a very low pH, zein could be solubilized by denaturation; on the other hand, a pH above the isoelectric point of the protein would increase electrostatic repulsion. For such, pH above the isoelectric result in a greater contact surface with the medium (water in this case), which in turn facilitates hydration and water absorption.^142^ The basic effect of pH on zein can also more easily ionize Ca­(H_2_PO_4_)_2_·H_2_O molecules into Ca^+^, H_2_PO_4_^–^ y HPO_4_^2–^.

## Conclusions

In this research, it was possible to develop zein nanoparticles loaded with monobasic phosphate monohydrate by nanoprecipitation; the particles were spherical with a uniform morphology. The results showed that zein and Ca­(H_2_PO_4_)_2_·H_2_O concentrations influence the size, polydispersity index, and potential Z of the nanoparticles. Also, FTIR and DSC tests confirmed the hydrogen bonding interaction of phosphate and zein groups. Furthermore, it was observed, through the deconvolution of amide I performed in the FTIR spectrum, that the zein nanoparticle is mainly composed of α-helices under the conditions described previously.

The results hint at a possible application to improve crop yield and nutrients efficiency. However, the current study had limitations such as the lack of tests for long-term stability of the nanoparticles and their performance in a wide range of environmental conditions. Nonetheless, future research could be aimed to optimize the synthesis conditions and to test the release of nutrients on agricultural crops. In conclusion, these findings help the development of sustainable fertilizers with potential application in precision agriculture by providing an alternative to improve nutrients absorption and reduce the negative effects of conventional fertilizers.

## Supplementary Material



## Notes

Infrared spectrum of zein, phosphate (Ca­(H_2_PO_4_)_2_··H_2_O), and poloxamer 188; experimental design of nanoparticles by nanoprecipitation; and experimental design of nanoparticles with phosphorus by nanoprecipitation (PDF)
