# Supplementary material for: Preparation and Characterization of Zein-Phosphate Nanoparticles by Nanoprecipitation Method with Potential Use as Fertilizer
Source: ACS Omega. 2025 Jun 13;10(24):25746–65. doi: 10.1021/acsomega.5c01817 (PMC12199041; doi:10.1021/acsomega.5c01817)
Supplement: Supplementary file 1 [file ao5c01817_si_001.pdf]

Preparation and characterization of zein-phosphate nanoparticles by nanoprecipitation method with potential use as fertilizer

Milagros Guadalupe Alvarez-Moreno[1]; *Francisco Rodríguez-Félix[1]; Carlos Gregorio Barreras-Urbina[2]; Maribel Plascencia-Jatomea[1]; Edgar Omar Rueda-Puente[3]; Juan José Reyes-Pérez[4]; José Agustín Tapia-Hernández[1]; Silvia Elena Burruel-Ibarra[5]; Tomás Jesús Madera-Santana[2]; Itzel Yanira López-Peña[2]; Josué Elías Juárez-Onofre[6]; Irela Santos-Sauceda[5].

[1] Departamento de Investigación y Posgrado en Alimentos, Universidad de Sonora, Blvd. Luis Encinas J, Calle Av. Rosales &, Centro, 83000 Hermosillo, Sonora, México.
[2] Centro de Investigación en Alimentación y Desarrollo, Carretera Gustavo Enrique Astiazarán Rosas, No. 46, Col. La Victoria, CP. 83304.
[3] Departamento de Agricultura y Ganadería, Universidad de Sonora, Carretera 100 a Bahia de Kino km. 21.5, Hermosillo, Sonora, México.
[4] Universidad Técnica Estatal de Quevedo, Av. Carlos J. Arosemena 38, Quevedo, Ecuador.
[5] Departamento de Investigación en Polímeros y Materiales, Universidad de Sonora.
[6] Departamento de Investigación en Física, Universidad de Sonora.

***Author for correspondence:** rodriguez_felix_fco@hotmail.com

Figure S1. Infrared spectrum of zein, phosphate ($Ca(H_2PO_4)_2 \cdot H_2O$), and poloxamer 188.

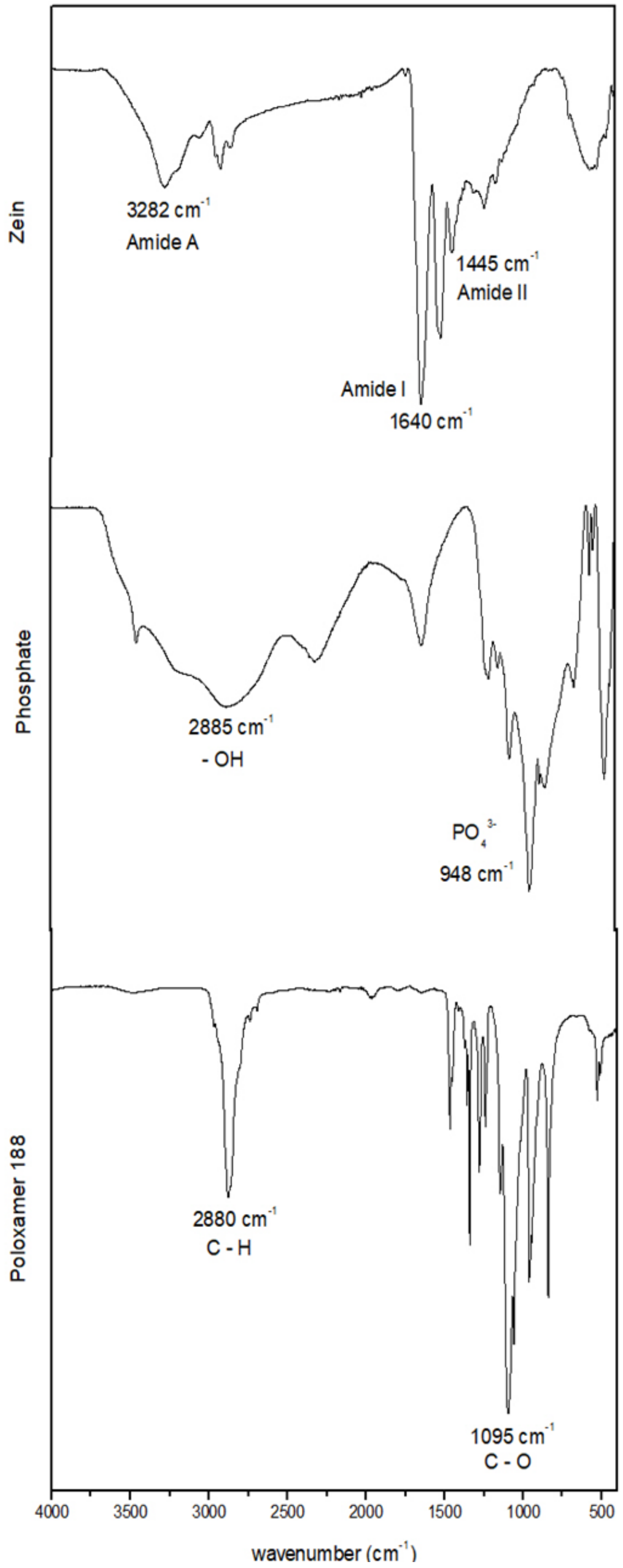

Table S1. Experimental design of nanoparticles by nanoprecipitation.

Zein (% w/v)	Ethanol (% v/v)	Poloxamer 188 (% w/v)	Agitation (rpm)	Flow rate (mL/h)
**2**	70	0.05	100	5
**2**	70	0.1	100	5
**2**	70	0.15	100	5
**3**	70	0.05	100	5
**3**	70	0.1	100	5
**3**	70	0.15	100	5
**4**	70	0.05	100	5
**4**	70	0.1	100	5
**4**	70	0.15	100	5

Table S2. Experimental design of nanoparticles with phosphorus by nanoprecipitation.

Zein (% w/v)	Ethanol (% v/v)	Poloxamer 188 (% w/v)	$[Ca(H_2PO_4)_2 \cdot H_2O]$ (% w/v)
**2**	70	0.05	1
**2**	70	0.05	2
**2**	70	0.05	3
**2**	70	0.1	1
**2**	70	0.1	2
**2**	70	0.1	3
**2**	70	0.15	1
**2**	70	0.15	2
**2**	70	0.15	3
**3**	70	0.05	1
**3**	70	0.05	2
**3**	70	0.05	3
**3**	70	0.1	1
**3**	70	0.1	2
**3**	70	0.1	3
**3**	70	0.15	1
**3**	70	0.15	2
**3**	70	0.15	3

**4**	70	0.05	1
**4**	70	0.05	2
**4**	70	0.05	3
**4**	70	0.1	1
**4**	70	0.1	2
**4**	70	0.1	3
**4**	70	0.15	1
**4**	70	0.15	2
**4**	70	0.15	3
